# The Association of Post-Materialism with Health Care Use. Findings of a General Population Survey in Germany

**DOI:** 10.3390/ijerph17238869

**Published:** 2020-11-28

**Authors:** André Hajek, Hans-Helmut König

**Affiliations:** Department of Health Economics and Health Services Research, University Medical Center Hamburg-Eppendorf, Martinistraße 52, 20246 Hamburg, Germany; h.koenig@uke.de

**Keywords:** Andersen’s behavioral model, health care use, health care utilization, materialism, physician visits, post-materialism, values, cultural values, health service use, health-promotion, screening, vaccination, check-up

## Abstract

(1) The aim of this study was to identify the association between post-materialism and health care use (in terms of the frequency of doctor visits and the reason for doctor visits). (2) Data were taken from the German General Social Survey (a representative sample of individuals aged 18 years and over, *n* = 3338). The Inglehart’s post-materialist index was used to quantify post-materialism. The doctor visits (self-reported) in the past three months served as an outcome measure. The reasons for seeing a doctor served as an additional outcome measure (acute illness; chronic illness; feeling unwell; requesting advice; visit to the doctor’s office without consulting the doctor (e.g., need to get a prescription); preventive medical check-up/vaccination). (3) After adjusting for several covariates, negative binomial regressions revealed that compared with materialism, post-materialism was associated with decreased doctor visits (total sample; women). Moreover, the likelihood of visiting the doctor for reasons of chronic illnesses was lower in post-materialistic women, whereas the likelihood of visiting the doctor for reasons of preventive medical check-up/vaccination was higher in post-materialistic women. (4) Study findings identify an unexplored link between post-materialism and doctor visits in women. One may conclude that in the long-term, the increased likelihood of preventive medical check-ups in post-materialistic women will be beneficial in decreasing the need for doctor visits for reasons of chronic illnesses. However, future research is required to elucidate the underlying mechanisms.

## 1. Introduction

To date, various studies have studied the determinants of health care use [1]. This can help to manage health care use, as well as avoid misuse (including under- and overuse). These studies mainly refer to the Andersen model [2], which distinguishes between predisposing characteristics (e.g., sex), enabling resources (e.g., income or perceived access to health care), and need factors (e.g., physical functioning or self-rated health). Systematic reviews demonstrated that need factors are particularly important for the use of health care services [1,3].

However, thus far, some factors are underexplored. For example, some recent studies showed that psychosocial factors [4] and personality characteristics [5,6] are also important for determining health care use. Moreover, another study [7] has investigated the link between *cultural factors* (among others, religious affiliation and ethnic identity) and health care use. However, we are only aware of one study that examined cultural values in terms of materialistic and post-materialistic values in this research area. This study focused on the use of complementary medicine as an outcome measure, showing that post-materialists used complementary medicine more frequently than materialists [8]. 

Based on the microdata available, there is a lack of studies on post-materialism and health care use in terms of doctor visits. In the same vein, there is a lack of studies examining connections between post-materialism and the reason for seeing a doctor. The term post-materialism goes back to Inglehart [9], who identified a shift in intergenerational cultural values among industrialized countries (among others, from survival orientation to self-expression or free speech) that can, for example, be explained by the affluence of the post-World War II period. To close this gap in knowledge, the purpose of this study was to identify the association between post-materialism and health care use (in terms of the frequency of doctor visits and the reason for doctor visits) in the total sample and stratified by sex—based on a nationally representative sample of the general adult population in Germany. Knowledge regarding the link between post-materialism and health care use may provide first insights into the link between cultural values (in terms of materialistic and post-materialistic values) and health care use. This may assist in better understanding the role cultural values have in the use of health care services. Ultimately, this may be beneficial for the management of health care use. Furthermore, knowledge about differences in the likelihood of visiting the doctor for reasons of preventive medical check-up/vaccination between materialistic and post-materialistic individuals may help to identify individuals at risk of potential underuse of these services.

It has been shown that post-materialism is associated with health-promoting behaviors and better health outcomes in high-income countries [10]. While it may sound illogical that a shift away from traditional values that focus more on survival may ultimately be beneficial for health-related outcomes, there are good arguments for it. Post-materialistic individuals are associated with health-promoting behaviors such as decreased smoking or decreased alcohol intake [10]. In line with this, we also assume that post-materialistic individuals have an increased use of preventive health care services. Various studies have demonstrated that health-related behaviors can contribute to the development of various chronic conditions such as stroke, diabetes, heart disease, and cancer [11]. An increase in chronic conditions is ultimately associated with increased health care use [12]. We therefore hypothesize that post-materialistic individuals have fewer doctor visits compared with materialistic individuals. Furthermore, we hypothesize that post-materialistic individuals have a decreased likelihood of visiting the doctor for reasons of chronic illnesses. In contrast, we hypothesize that post-materialistic individuals have an increased likelihood of visiting the doctor for reasons of preventive medical check-up/vaccination. With regard to sex-stratified regressions, we do not have any prespecified hypotheses.

## 2. Materials and Methods

### 2.1. Sample

For our study, cross-sectional data were taken from the German General Social Survey (“ALLBUS”). Starting in the year 1980, the surveys of the ALLBUS study have been conducted every second year. These are nationally representative samples (random samples) from individuals ≥18 years residing in private households. The samples of the ALLBUS study have been extended to include German-speaking foreigners (and residing in Germany) as well as to the former East Germany. Commonly, more than 3000 individuals participate in the ALLBUS survey. The ALLBUS data are often used for social science research purposes in Germany. Various topics are included in the ALLBUS survey, such as different attitudes or opinions, as well as general demographic data. Data were collected using computer-assisted personal interviews. Terwey [13] provides additional details regarding the ALLBUS surveys.

Special attention was paid to health factors like doctor visits in the 2014 ALLBUS survey (which took place from March to September; *n* = 3471 individuals were interviewed). This is the reason why we restricted our current study to this wave [14]. With refusal as a key reason for nonparticipation, the response rate was 35% in this specific wave. Similar response rates have also been detected in other survey studies performed in Germany [15]. This is also in line with the trend of declining rates of participation in Germany [16]. Additional details are given in the strengths and limitations section. 

### 2.2. Outcome Variables

Our main outcome measure was the number of doctor visits (self-reported) in the past three months. Individuals should refer to “visits to doctor’s surgeries and outpatient treatments in clinics and casualty departments, but not examinations during an inpatient stay in a hospital or accompanying relatives or children to the doctor” [17]. This includes all kinds of outpatient physician visits (including dental visits). 

For individuals with at least one doctor visit, the reason for seeing a doctor in the preceding three months was also included [17]:

- Acute illness (e.g., flu, injury);
- Chronic illness (e.g., diabetes, high blood pressure/hypertension, rheumatism);
- Felt unwell (e.g., general discomfort, sleep disorders);
- Requesting advice;
- Visit to the doctor’s office, but without consulting the doctor (e.g., need to get a prescription, radiotherapy);
- Preventive medical check-up/vaccination.

The individuals could choose between no and yes (multiple responses were possible).

### 2.3. Key Independent Variable

The four items for post-materialism [18] were introduced as follows [17]:

In politics too one cannot have everything at once. On this card are four goals which can be pursued in politics. If you had to choose between these different goals, which one would seem to you personally to be the most important? And which goal would be the second most important to you? And which goal would be third? And which goal would be fourth? Only one response possible for each item.

The items were: 

A To maintain law and order in this country; 

B To give citizens more influence on government decisions;

C To fight rising prices; 

D To protect the right of freedom of speech.

Materialist values indicate economic or physical insecurity (A and C). When individuals chose A and C as the two most important goals, they were classified as “materialists”. When individuals chose B and D (post-bourgeois/post-materialist value orientations) as the two most important goals, they were classified as “post-materialists” [18]. All other cases were classified as “mixed type” [19]: when individuals chose B or D as the most important one and A or C as the second most important one, they were classified as “post-materialistic mixed type”. When individuals chose A or C as the most important one and B or D as the second most important one, they were classified as “materialistic mixed type”. 

### 2.4. Covariates

Drawing on Andersen’s behavioral model [2], covariates were chosen [20]. Predisposing characteristics were included as follows: sex, age, family status (0 = married and living apart; widowed; divorced; never married; civil partnership, living apart; registered partner deceased; civil partnership dissolved; 1 = married and living together with spouse; civil partnership, living together), education (ISCED-97 [21]): basic education, lower secondary, upper secondary, post-secondary, higher tertiary, and upper tertiary). Covariates related to enabling resources, such as perceived access to health care use, were not included for reasons of data availability.

With regard to need factors, the number of chronic conditions (allergy; migraine; high blood pressure, hypertension; circulatory disorder of the heart, angina pectoris; rheumatism, chronic inflammation of the joints, arthritis, arthrosis, gout; spinal damage; chronic bronchitis; asthma; inflammation of the liver, hepatitis, liver shrinkage, liver cirrhosis; diabetes; cancer; osteoporosis) were included. Furthermore, impairments in activities of daily living (1. when climbing stairs; 2. in coping with everyday tasks such as lifting something heavy) were included (from 1 = health affects you not at all when performing these activities to 3 = health affects you greatly when performing these activities). Furthermore, body mass index (BMI) categories (underweight: BMI < 18.5 kg/m^2^, normal weight: 18.5 kg/m^2^ ≤ BMI < 25 kg/m^2^, overweight: 25 kg/m^2^ ≤ BMI < 30 kg/m^2^, and obesity: BMI ≥ 30 kg/m^2^) and smoking behavior (no/yes) were included as lifestyle factors. 

In sensitivity analyses, the model was additionally adjusted for covariates including seasonal effects (monthly dummies), the predisposing characteristic employment status (currently employed; currently not employed), and the enabling resources town size (1–1999 inhabitants; 2000–4999 inhabitants; 5000–19,999 inhabitants; 20,000–49,999 inhabitants; 50,000–99,999 inhabitants, 100,000–499,999 inhabitants; ≥500,000 inhabitants) and (log) household net equivalent income. Moreover, age-stratified regression models were presented. 

### 2.5. Statistical Analysis

Sample characteristics were reported first. Subsequently, multiple negative binomial regressions (with robust standard errors) were used to examine the association between post-materialism and the frequency of doctor visits in the past three months (total sample and stratified by sex). Additionally, the association between post-materialism and the reason for doctor visits (outcome measures: reason for doctor visits: acute illness; chronic illness; felt unwell; requesting advice; visit to the doctor’s office, but without consulting the doctor; preventive medical check-up/vaccination) was examined using multiple logistic regressions (stratified by sex) [20].

The level of significance was set at α = 0.05. Statistical analysis was conducted using Stata 16.0 (Stata Corp., College Station, TX, USA).

## 3. Results

### 3.1. Sample Characteristics

Stratified by sex, Table 1 displays the characteristics of our sample for the individuals included in regression analysis. In total, average age equaled 49.7 years (SD: 17.4 years; from 18 to 91 years) and 48.7% of the individuals were female. 

In men, 27.8% of the individuals were classified as post-materialists, and in women, 28.9% of the individuals were classified as post-materialists. The mean number of doctor visits was 1.8 (SD: 2.8) in men and 2.4 (SD: 3.8) in women. The most frequent reasons for doctor visits were “acute illness”, “chronic illness” and “preventive medical check-up/vaccination”. Additional details are given in Table 1.

It may be worth noting that when stratified by values, materialists had on average 1.4 chronic diseases (SD: 1.5), whereas post-materialists had on average 1.0 chronic diseases (SD: 1.4). These differences were significant (*p* < 0.001). 

In Supplementary Table S1, the analytical sample is displayed stratified by our key independent variable (materialism/post-materialism status) and sex. Particularly differences in health-related factors (e.g., number of chronic diseases, *p* = 0.016) were present when comparing female materialists with female post-materialists. Please see Supplementary Table S1 for further details. 

### 3.2. Regression Analysis

Compared to Poisson models (see Supplementary Table S2), the negative binomial regressions had smaller AIC and BIC values (Table 2), suggesting that it fits our data better. 

The determinants of doctor visits in the past three months are displayed in Table 2 (total sample; men; women). The control variables are not shown in Table 2 and Table 3 for the sake of clarity (the coefficients for the covariates are shown in Supplementary Tables S3 and S4). In Supplementary Tables S5 and S6, the results are displayed with “Post-materialist” as reference category. After adjusting for several covariates, negative binomial regressions showed that the number of doctor visits was lower in post-materialists compared with materialists in the total sample (incidence rate ratio (IRR): 0.83 (95% confidence interval (CI): 0.70–0.99) and in women (IRR: 0.72 (95% CI: 0.57–0.89)). Moreover, they showed that the number of doctor visits was lower in female post-materialistic mixed types compared with female materialists (IRR: 0.80 (95% CI: 0.65–0.99).

With regard to covariates, only the number of chronic conditions and impairments in activities of daily living were consistently associated with an increased number of doctor visits in the total sample and in both sexes (see Supplementary Table S3).

The reason for seeing a doctor in the preceding three months was documented for individuals reporting one or more doctor visits in this period (only stratified by sex—for reasons of clarity). The determinants of six outcome variables are displayed in Table 3 (reason: (1) acute illness, (2) chronic illness, (3) feeling unwell, (4) requesting advice, (5) visit to the doctor’s office, but without consulting the doctor, (6) preventive medical check-up/vaccination). Multiple logistic regressions showed that the likelihood of visiting the doctor for reasons of chronic illnesses was lower in post-materialistic women compared with materialistic women (OR: 0.48 (95% CI: 0.29–0.79), whereas the likelihood of visiting the doctor for reasons of preventive medical check-up/vaccination was higher in post-materialistic women compared with materialistic women (OR: 1.64 (95% CI: 1.02–2.64). Similarly, compared to materialistic women, the likelihood of visiting the doctor for reasons of chronic illnesses was lower in female materialistic mixed types (OR: 0.60 (95% CI: 0.38–0.96) and female post-materialistic mixed types (OR: 0.44 (95% CI: 0.28–0.71). Compared to materialistic women, the likelihood of visiting the doctor for reasons of preventive medical check-up/vaccination was higher in female materialistic mixed types (OR: 1.79 (95% CI: 1.12–2.84) and female post-materialistic mixed types (OR: 2.25 (95% CI: 1.43–3.56).

With regard to the covariates, the number of chronic conditions was, among other things, associated with doctor visits for reasons of “chronic illnesses”, or “visit to the doctor’s office, but without consulting the doctor” in men and in women (see Supplementary Table S4 for further details regarding the covariates).

In sensitivity analysis, the model was additionally adjusted for (log) household net equivalent income. Our key findings remained almost the same (see Supplementary Tables S7 and S8). In another sensitivity analysis, the model was additionally adjusted for employment status. Again, our main results remained nearly identical (see Supplementary Tables S9 and S10). In further sensitivity analysis, the main model was extended by adding monthly dummies, which produced virtually the same results (see Supplementary Tables S11 and S12). In another sensitivity analysis, the main model was extended by adding town size (see Supplementary Tables S13 and S14). Again, the findings did not change substantially. In a last sensitivity analysis, regressions were also calculated stratified by age group (median split: the first group included individuals aged 18 to 49 years and the second group included individuals aged 50 to 91 years; see Supplementary Table S15). The link between post-materialism and the number of doctor visits remained similar in younger and older women. However, it should be noted that there were additional differences between younger female materialists and materialistic as well as post-materialistic mixed types. Moreover, some differences appeared between younger male materialists and materialistic as well as post-materialistic mixed types.

## 4. Discussion

### 4.1. Main Findings

Based on a large nationally representative sample, the aim of this cross-sectional study was to identify the association between post-materialism and health care use (in terms of the frequency of doctor visits and the reason for doctor visits). After adjusting for various covariates, negative binomial regressions revealed that, compared with materialism, post-materialism was associated with decreased doctor visits (total sample; women). Interestingly, the likelihood of visiting the doctor for reasons of chronic illnesses was lower in post-materialistic women, whereas the likelihood of visiting the doctor for reasons of preventive medical check-up/vaccination was higher in post-materialistic women.

### 4.2. Possible Explanations

This is the first study analyzing the link between post-materialism and doctor visits, as well as the reason for seeing a doctor, and therefore extends our current knowledge. This study supports research that aims to develop a better understanding of the role cultural values have in health care use. However, while our study produced the first results regarding the link between post-materialism and doctor visits, future research (e.g., in different countries) is required to examine this link in more detail. More advanced analytical approaches could be used in future studies.

Due to the lack of studies in this area, it is difficult to compare our study with previous findings. Nevertheless, we have some possible explanations for our findings: We assume that the link between post-materialistic values and decreased doctor visits can be explained by general lifestyle and health-related behavior of these post-materialistic individuals. The association between post-materialistic values and health-related behavior is supported by a study conducted by Mackenbach [10]. In the long term, these health-related and health promotion (such as using preventive health care services) behaviors may be associated with decreased need factors [11], which are in turn associated with decreased health care use [12].

This explanation is in line with our additional findings. We found that the likelihood of visiting the doctor for reasons of chronic illnesses was lower in post-materialistic women, whereas the likelihood of visiting the doctor for reasons of preventive medical check-up/vaccination was higher in post-materialistic women.

It should be stressed that several need factors were adjusted for in our study. Therefore, we assume that other factors may also be important to explain the link between post-materialism and, for example, the likelihood of vising the doctor for preventive care. We assume that post-materialistic individuals may have differing attitudes towards doctors than those of materialistic individuals. More precisely, we assume that they may be more open towards, and tolerant of, doctor visits, particularly preventive doctor visits. In line with this, the proportion of individuals who refuse to see a doctor for reasons of preventive medical check-up/vaccination may be smaller. We think that our results regarding differences between female materialists and female (post-)materialistic mixed types can also be explained by these aforementioned factors. Nevertheless, future research is required to test these assumptions. 

However, it should also be noted that other, somewhat contrary, findings exist for health promotion activities such as vaccination [22]. For instance, based on a data from the West Coast survey, it has been shown that antivaccination positions are associated with post-materialist values [22]. This means that individuals are more likely to support antivaccination positions when they identify as a post-materialist. Steel and Wolters [22] stated that post-materialist values are associated with being more “liberal” and favor antiwar and antinuclear activism, civil rights, gender equality, and the protection of the environment. Therefore, they assume that these post-materialism’s liberal tendencies (i.e., civil liberties) and the desire to make one’s own decisions may drive antivaccination positions. However, future research in this area is required [22].

It is quite puzzling why the likelihood of visiting the doctor for reasons of chronic illnesses was lower in post-materialistic women (as well as in both mixed types). A possible explanation may be that there are differences in the severity of chronic diseases. However, this should be investigated in future studies. For example, different approaches could be used in future studies (e.g., distinguishing between low and high users).

Future research is required to clarify why there was only a link in women, but not in men. We assume that this may be explained by the fact that there are more preventive health services available for women (for example, for pap smears or mammography). Another possible explanation for the nonsignificant association among men may be that the materialistic male subsample is rather small (i.e., lack of statistical power).

### 4.3. Strengths and Limitations

This is the first study investigating the association between cultural values, in terms of materialism/post-materialism, and health care use. A large nationally representative sample was used in our study. Since the recall period for doctor visits was rather low, a small potential recall bias may be possible [23]. It should be emphasized that the reasons for doctor visits were also examined. 

The potential for sample selection bias should be acknowledged as the response rate is rather low in the ALLBUS study. Nevertheless, efforts were undertaken to mitigate this potential bias in the ALLBUS study [24]. From a theoretical perspective, post-materialistic values are considered as quite stable over time. However, it has been shown that these values can actually change within individuals over time [25]. Therefore, future longitudinal studies are required to gain an understanding of the link between post-materialism and health care use over time. Similarly, it should be acknowledged that the four-item scale is sensitive to short-term influences. For example, factors like temporarily high inflation can affect this four-item scale [25]. Therefore, future research with the 12-item scale developed by Inglehart [9] may be beneficial. It should be acknowledged that enabling resources such as having a supplementary private insurance were not included in the ALLBUS study.

## 5. Conclusions

This study’s findings highlight an unexplored link between post-materialism and doctor visits in women. However, future research is required to elucidate the underlying mechanisms. Moreover, it should be stressed that these are preliminary results. Further work is required (e.g., with different model specifications and based on different analytical approaches such as latent class approaches or finite mixture models [26,27]).

## Figures and Tables

**Table 1:** Sample characteristics for the individuals included in negative binomial regressions stratified by sex (*n* = 3338).

	Men (*n* = 1711)	Women (*n* = 1627)
Age: Mean (SD); Range	49.8 (17.5); 18–91	49.6 (17.3); 18–91
Married, living together with spouse/partner: N (%)	991 (42.1%)	879 (54.0%)
Education (ISCED-97): N (%)		
Basic education	17 (1.0%)	24 (1.5%)
Lower secondary	112 (6.6%)	179 (11.0%)
Upper secondary	810 (47.3%)	762 (46.8%)
Post-secondary	91 (5.3%)	143 (8.8%)
Higher tertiary	638 (37.3%)	498 (30.6%)
Upper tertiary	43 (2.5%)	21 (1.3%)
Weight category: N (%)		
Underweight	13 (0.8%)	51 (3.1%)
Normal weight	639 (37.3%)	817 (50.2%)
Overweight	724 (42.3%)	487 (30.0%)
Obese	335 (19.6%)	272 (16.7%)
Currently smoking: N (%)	568 (33.2%)	393 (24.2%)
Number of chronic diseases: Mean (SD); Range	1.1 (1.2); 0–9	1.3 (1.4); 0–9
Activities of daily living (climbing stairs): N (%)		
Not at all affected	1160 (67.8%)	990 (60.8%)
Slightly affected	381 (22.3%)	434 (26.7%)
Greatly affected	170 (9.9%)	203 (12.5%)
Activities of daily living (coping with everyday tasks): N (%)		
Not at all affected	1009 (59.0%)	872 (53.6%)
Slightly affected	475 (27.7%)	489 (30.1%)
Greatly affected	227 (13.3%)	266 (16.3%)
Materialism and post-materialism: N (%)		
Materialist	149 (8.7%)	202 (12.4%)
Materialistic mixed type	513 (30.0%)	441 (27.1%)
Post-materialistic mixed type	574 (33.5%)	514 (31.6%)
Post-materialist	475 (27.8%)	470 (28.9%)
Number of doctor visits: Mean (SD); Range; Proportion of individuals with no doctor visits	1.8 (2.8); 0–48; 31.9%	2.4 (3.8); 0–65; 25.5%
Reason for doctor visit (yes): Acute illness: N (%)	350 (29.9%)	380 (31.3%)
Reason for doctor visit (yes): Chronic illness: N (%)	328 (28.0%)	380 (31.3%)
Reason for doctor visit (yes): Felt unwell: N (%)	77 (6.6%)	116 (9.6%)
Reason for doctor visit (yes): Requesting advice: N (%)	151 (12.9%)	143 (11.8%)
Reason for doctor visit (yes): visit to the doctor’s office, but without consulting the doctor: N (%)	205 (17.5%)	250 (20.6%)
Reason for doctor visit (yes): preventive medical check-up/vaccination: N (%)	315 (26.9%)	397 (32.7%)

Note: The reasons for doctor visit sum to 1170 (men) and 1214 (women).

**Table 2:** Determinants of frequency of physician visits (total sample and stratified by sex). Results of negative binomial regressions.

	(1)	(3)	(5)
	Frequency of Physician Visits—Total Sample	Frequency of Physician Visits—Men	Frequency of Physician Visits—Women
Materialistic mixed type (Reference category: Materialist)	0.95	1.18	0.87
	(0.81–1.13)	(0.93–1.50)	(0.70–1.09)
Post-materialistic mixed type	0.89	1.14	0.80 *
	(0.75–1.05)	(0.90–1.45)	(0.65–0.99)
Post-materialist	0.83 *	1.10	0.72 **
	(0.70–0.99)	(0.87–1.39)	(0.57–0.89)
Covariates	√	√	√
Constant	1.10	0.65 *	1.70 **
	(0.85–1.43)	(0.47–0.91)	(1.18–2.45)
Observations	3338	1711	1627
Pseudo R^2^	0.053	0.058	0.056
AIC	12,284.89	5903.13	6335.18
BIC	12413.26	6017.47	6448.47
k	27	27	27
df	21	21	21

Incidence rate ratios are reported; confidence intervals in parentheses; ** *p* < 0.01, * *p* < 0.05; Covariates include age, marital status, education, number of impairments, chronic conditions, in activities of daily living, smoking behavior, and weight category.

**Table 3:** Determinants of seeing the doctor (for several reasons). Results of logistic regressions (1 = yes, visiting the doctor for this reason; 0 = otherwise).

	Reason for Doctor Visit: Acute Illness—Men	Reason for Doctor Visit: Chronic Illness—Men	Reason for Doctor Visit: Felt Unwell—Men	Reason for Doctor Visit: Requesting Advice—Men	Reason for Doctor Visit: Visit to the Doctor’s Office (without Consulting the Doctor)—Men	Reason for Doctor Visit: Preventive Medical Check-Up/Vaccination—Men	Reason for Doctor Visit: Acute Illness—Women	Reason for Doctor Visit: Chronic Illness—Women	Reason for Doctor Visit: Felt Unwell—Women	Reason for Doctor Visit: Requesting Advice—Women	Reason for Doctor Visit: Visit to the Doctor’s Office (without Consulting the Doctor)—Women	Reason for Doctor Visit: Preventive Medical Check-Up/Vaccination—Women
Materialistic mixed type (Reference category: Materialist)	1.49	1.30	0.86	0.59	0.74	0.94	1.14	0.60 *	0.82	1.17	1.23	1.79 *
	(0.85–2.62)	(0.74–2.29)	(0.35–2.09)	(0.31–1.11)	(0.42–1.30)	(0.56–1.59)	(0.73–1.76)	(0.38–0.96)	(0.45–1.49)	(0.66–2.09)	(0.76–2.00)	(1.12–2.84)
Post-materialistic mixed type	1.31	1.20	0.79	0.69	0.64	0.79	1.05	0.44 ***	0.76	0.93	1.35	2.25 ***
	(0.74–2.30)	(0.69–2.11)	(0.32–1.93)	(0.37–1.28)	(0.36–1.12)	(0.47–1.34)	(0.68–1.61)	(0.28–0.71)	(0.42–1.39)	(0.52–1.68)	(0.83–2.17)	(1.43–3.56)
Post-materialist	1.15	1.22	1.10	0.80	0.77	0.92	1.24	0.48 **	0.60	1.07	1.18	1.64 *
	(0.64–2.05)	(0.68–2.20)	(0.44–2.75)	(0.42–1.52)	(0.43–1.39)	(0.54–1.58)	(0.80–1.94)	(0.29–0.79)	(0.31–1.15)	(0.59–1.97)	(0.71–1.95)	(1.02–2.64)
Covariates	√	√	√	√	√	√	√	√	√	√	√	√
Constant	2.75 *	0.03 ***	0.04 ***	0.15 ***	0.05 ***	0.20 ***	1.95 +	0.05 ***	0.22 **	0.08 ***	0.07 ***	0.21 ***
	(1.27–5.98)	(0.01–0.07)	(0.01–0.17)	(0.06–0.41)	(0.02–0.13)	(0.09–0.44)	(0.96–3.98)	(0.02–0.12)	(0.08–0.63)	(0.03–0.23)	(0.03–0.17)	(0.10–0.45)
Observations	1170	1170	1170	1159	1163	1163	1214	1214	1214	1214	1214	1214
Pseudo R^2^	0.094	0.179	0.040	0.033	0.086	0.033	0.057	0.267	0.025	0.015	0.042	0.041

Incidence rate ratios are reported; confidence intervals in parentheses; *** *p* < 0.001, ** *p* < 0.01, * *p* < 0.05, + *p* < 0.10; Covariates include age, marital status, education, number of impairments, chronic conditions, in activities of daily living, smoking behavior, and weight category.

## Supplementary Materials

The following are available online at https://www.mdpi.com/1660-4601/17/23/8869/s1, Table S1: Sample characteristics for the individuals included in negative binomial regressions stratified by sex and materialism/post-materialism status (*n* = 3338), Table S2: Determinants of frequency of physician visits (total sample and stratified by sex). Results of Poisson regressions, Table S3: Determinants of frequency of physician visits (total sample and stratified by sex). Results of negative binomial regressions (also displaying the coefficients for the covariates), Table S4: Determinants of seeing the doctor (for several reasons). Results of logistic regressions (1 = yes, visiting the doctor for this reason; 0 = otherwise) (also displaying the coefficients for the covariates), Table S5: Determinants of frequency of physician visits (total sample and stratified by sex). Results of negative binomial regressions (with ‘Post-materialist’ as reference category), Table S6: Determinants of seeing the doctor (for several reasons). Results of logistic regressions (1 = yes, visiting the doctor for this reason; 0 = otherwise) (with ‘Post-materialist’ as reference category), Table S7: Determinants of frequency of physician visits (total sample and stratified by sex). Results of negative binomial regressions (main model extended by (log) household net equivalent income), Table S8: Determinants of seeing the doctor (for several reasons). Results of logistic regressions (1 = yes, visiting the doctor for this reason; 0 = otherwise) (main model extended by (log) household net equivalent income), Table S9: Determinants of frequency of physician visits (total sample and stratified by sex). Results of negative binomial regressions (main model extended by employment status), Table S10: Determinants of seeing the doctor (for several reasons). Results of logistic regressions (1 = yes, visiting the doctor for this reason; 0 = otherwise) (main model extended by employment status), Table S11: Determinants of frequency of physician visits (total sample and stratified by sex). Results of negative binomial regressions (main model extended by monthly dummies), Table S12: Determinants of seeing the doctor (for several reasons). Results of logistic regressions (1 = yes, visiting the doctor for this reason; 0 = otherwise) (main model extended by monthly dummies), Table S13: Determinants of frequency of physician visits (total sample and stratified by sex). Results of negative binomial regressions (main model extended by town size), Table S14: Determinants of seeing the doctor (for several reasons). Results of logistic regressions (1 = yes, visiting the doctor for this reason; 0 = otherwise) (main model extended by town size), Table S15: Determinants of frequency of physician visits (total sample and stratified by sex). Results of negative binomial regressions (main model stratified by age group).
