# Supplementary material for: The Association of Post-Materialism with Health Care Use. Findings of a General Population Survey in Germany
Source: Int J Environ Res Public Health. 2020 Nov 28;17(23):8869. doi: 10.3390/ijerph17238869 (PMC7730980; doi:10.3390/ijerph17238869)
Supplement: Supplementary file 1 [file ijerph-17-08869-s001.pdf]

Table S1. Sample characteristics for the individuals included in negative binomial regressions stratified by sex and materialism/post-materialism status (*n* = 3338).

Variables	Men					Women				
	Materialist	Materialistic Mixed Type	Post-Materialistic Mixed Type	Post-Materialist	*P*-value[2]	Materialist	Materialistic Mixed Type	Post-Materialistic Mixed Type	Post-Materialist	*P*-value
	N = 149	*N* = 513	*N* = 574	*N* = 475		*N* = 202	*N* = 441	*N* = 514	*N* = 470	
Age: Mean (SD)	52.0 (19.4)	51.5 (18.5)	50.0 (17.0)	46.9 (16.0)	0.03	53.5 (20.1)	50.5 (17.7)	50.0 (16.5)	46.6 (16.0)	0.03
Marital status: N (%)					<0.01					0.41
Married, living together with spouse/partner	84 (56.4%)	305 (59.5%)	348 (60.6%)	254 (53.5%)		104 (51.5%)	251 (56.9%)	292 (56.8%)	232 (49.4%)	
Other[1]	65 (43.6%)	208 (40.5%)	226 (39.4%)	221 (46.5%)		98 (48.5%)	190 (43.1%)	222 (43.2%)	238 (50.6%)	
Education (ISCED-97): N (%)					0.39					0.03
- Basic education	5 (3.4%)	6 (1.2%)	4 (0.7%)	2 (0.4%)		7 (3.5%)	5 (1.1%)	8 (1.6%)	4 (0.9%)	
- Lower secondary	22 (14.8%)	41 (8.0%)	30 (5.2%)	19 (4.0%)		47 (23.3%)	56 (12.7%)	52 (10.1%)	24 (5.1%)	
- Upper secondary	76 (51.0%)	262 (51.1%)	275 (47.9%)	197 (41.5%)		107 (53.0%)	216 (49.0%)	247 (48.1%)	192 (40.9%)	
- Post secondary	5 (3.4%)	24 (4.7%)	29 (5.1%)	33 (6.9%)		11 (5.4%)	35 (7.9%)	47 (9.1%)	50 (10.6%)	
- Higher tertiary	36 (24.2%)	168 (32.7%)	225 (39.2%)	209 (44.0%)		29 (14.4%)	125 (28.3%)	154 (30.0%)	190 (40.4%)	
- Upper tertiary	5 (3.4%)	12 (2.3%)	11 (1.9%)	15 (3.2%)		1 (0.5%)	4 (0.9%)	6 (1.2%)	10 (2.1%)	
Weight category: N (%)					0.13					0.37
- Underweight	2 (1.3%)	5 (1.0%)	3 (0.5%)	3 (0.6%)		7 (3.5%)	9 (2.0%)	17 (3.3%)	18 (3.8%)	
- Normal weight	56 (37.6%)	189 (36.8%)	202 (35.2%)	192 (40.4%)		85 (42.1%)	217 (49.2%)	260 (50.6%)	255 (54.3%)	
- Overweight	60 (40.3%)	217 (42.3%)	249 (43.4%)	198 (41.7%)		63 (31.2%)	132 (29.9%)	156 (30.4%)	136 (28.9%)	
- Obese	31 (20.8%)	102 (19.9%)	120 (20.9%)	82 (17.3%)		47 (23.3%)	83 (18.8%)	81 (15.8%)	61 (13.0%)	
Currently smoking: N (%)					0.52					0.28
Yes	58 (38.9%)	169 (32.9%)	192 (33.4%)	149 (31.4%)		45 (22.3%)	97 (22.0%)	130 (25.3%)	121 (25.7%)	
No	91 (61.1%)	344 (67.1%)	382 (66.6%)	326 (68.6%)		157 (77.7%)	344 (78.0%)	384 (74.7%)	349 (74.3%)	
Number of chronic diseases: Mean (SD)	1.1 (1.2)	1.2 (1.4)	1.1 (1.2)	1.0 (1.1)	0.35	1.7 (1.7)	1.4 (1.4)	1.4 (1.5)	1.1 (1.2)	0.02
Activities of daily living (climbing stairs): N (%)					0.16					<0.01
Not at all affected	89 (59.7%)	343 (66.9%)	369 (64.3%)	359 (75.6%)		96 (47.5%)	260 (59.0%)	311 (60.5%)	323 (68.7%)	
Slightly affected	41 (27.5%)	118 (23.0%)	137 (23.9%)	85 (17.9%)		59 (29.2%)	122 (27.7%)	147 (28.6%)	106 (22.6%)	
Greatly affected	19 (12.8%)	52 (10.1%)	68 (11.8%)	31 (6.5%)		47 (23.3%)	59 (13.4%)	56 (10.9%)	41 (8.7%)	
Activities of daily living (coping with everyday tasks): *N* (%)					0.09					<0.001
Not at all affected	82 (55.0%)	291 (56.7%)	324 (56.4%)	312 (65.7%)		88 (43.6%)	216 (49.0%)	271 (52.7%)	297 (63.2%)	

Slightly affected	41 (27.5%)	149 (29.0%)	168 (29.3%)	117 (24.6%)		60 (29.7%)	144 (32.7%)	159 (30.9%)	126 (26.8%)	
Greatly affected	26 (17.4%)	73 (14.2%)	82 (14.3%)	46 (9.7%)		54 (26.7%)	81 (18.4%)	84 (16.3%)	47 (10.0%)	
Number of doctor visits: Mean (SD)	1.7 (3.0)	1.9 (2.7)	1.8 (3.1)	1.6 (2.2)	0.32	3.0 (3.9)	2.6 (4.7)	2.3 (3.4)	1.9 (3.2)	0.17
Reason for doctor visit: Acute illness: *N* (%)					0.26					0.45
No	75 (77.3%)	247 (69.0%)	277 (70.1%)	220 (69.6%)		118 (72.4%)	232 (69.5%)	267 (70.1%)	215 (64.6%)	
Yes	22 (22.7%)	111 (31.0%)	118 (29.9%)	96 (30.4%)		45 (27.6%)	102 (30.5%)	114 (29.9%)	118 (35.4%)	
Reason for doctor visit: Chronic illness: *N* (%)					0.03					0.01
No	69 (71.1%)	246 (68.7%)	282 (71.4%)	241 (76.3%)		82 (50.3%)	220 (65.9%)	274 (71.9%)	255 (76.6%)	
Yes	28 (28.9%)	112 (31.3%)	113 (28.6%)	75 (23.7%)		81 (49.7%)	114 (34.1%)	107 (28.1%)	78 (23.4%)	
Reason for doctor visit: Felt unwell: *N* (%)					0.05					0.12
No	90 (92.8%)	335 (93.6%)	373 (94.4%)	292 (92.4%)		143 (87.7%)	300 (89.8%)	345 (90.6%)	308 (92.5%)	
Yes	7 (7.2%)	23 (6.4%)	22 (5.6%)	24 (7.6%)		20 (12.3%)	34 (10.2%)	36 (9.4%)	25 (7.5%)	
Reason for doctor visit: Requesting advice: *N* (%)					0.08					0.16
No	80 (82.5%)	318 (88.8%)	345 (87.3%)	272 (86.1%)		143 (87.7%)	290 (86.8%)	341 (89.5%)	294 (88.3%)	
Yes	17 (17.5%)	40 (11.2%)	50 (12.7%)	44 (13.9%)		20 (12.3%)	44 (13.2%)	40 (10.5%)	39 (11.7%)	
Reason for doctor visit: visit to the doctor’s office, but without consulting the doctor: *N* (%)					0.32					0.71
No	73 (75.3%)	290 (81.0%)	332 (84.1%)	266 (84.2%)		131 (80.4%)	263 (78.7%)	296 (77.7%)	271 (81.4%)	
Yes	24 (24.7%)	68 (19.0%)	63 (15.9%)	50 (15.8%)		32 (19.6%)	71 (21.3%)	85 (22.3%)	62 (18.6%)	
Reason for doctor visit: preventive medical check-up/vaccination: *N* (%)					0.85					0.13
No	71 (73.2%)	260 (72.6%)	295 (74.7%)	226 (71.5%)		132 (81.0%)	227 (68.0%)	235 (61.7%)	220 (66.1%)	
Yes	26 (26.8%)	98 (27.4%)	100 (25.3%)	90 (28.5%)		31 (19.0%)	107 (32.0%)	146 (38.3%)	113 (33.9%)	

[1]: married and living apart; widowed; divorced; never married; civil partnership, living apart; registered partner deceased; civil partnership dissolved; [2]: Comparison based on oneway ANOVA or Chi-square test, as appropriate.

Table S2. Determinants of frequency of physician visits (total sample and stratified by sex). Results of Poisson regressions.

	(1)	(2)	(3)
	Frequency of Physician Visits–Total Sample	Frequency of Physician Visits–Men	Frequency of Physician Visits–Women
Materialistic mixed type (Reference category: Materialist)	1.00	1.12	0.95
	(0.83–1.19)	(0.84–1.48)	(0.75–1.21)
Post-materialistic mixed type	0.91	1.10	0.85
	(0.76–1.08)	(0.83–1.47)	(0.68–1.07)
Post-materialist	0.87	1.07	0.78*
	(0.72–1.04)	(0.81–1.42)	(0.61–0.99)
Covariates	✓	✓	✓
Constant	1.23	0.75	1.88**
	(0.93–1.63)	(0.52–1.07)	(1.24–2.84)
Observations	3338	1711	1627
Pseudo R^2	0.120	0.115	0.140
AIC	14,747.48	6778.58	7754.31
BIC	14,869.75	6887.47	7862.20
k	26	26	26
df	20	20	20

Incidence rate ratios were reported; confidence intervals in parentheses; *** p<0.001, ** p<0.01, * p<0.05, + p<0.10; Covariates include age, marital status, education, chronic conditions, number of impairments in activities of daily living, smoking behavior, and weight category.

Table S3. Determinants of frequency of physician visits (total sample and stratified by sex). Results of negative binomial regressions (also displaying the coefficients for the covariates)

	(1)	(2)	(3)
	Frequency of Physician Visits–Total Sample	Frequency of Physician Visits–Men	Frequency of Physician Visits–Women
Materialistic mixed type (Reference category: Materialist)	0.95	1.18	0.87
	(0.81–1.13)	(0.93–1.50)	(0.70–1.09)
Post-materialistic mixed type	0.89	1.14	0.80*
	(0.75–1.05)	(0.90–1.45)	(0.65–0.99)
Post-materialist	0.83*	1.10	0.72**
	(0.70–0.99)	(0.87–1.39)	(0.57–0.89)
Covariates:			
Age	1.00+	1.00+	0.99***
	(0.99–1.00)	(1.00–1.01)	(0.99–1.00)
Marital status:			
Married, living together with spouse/partner (Reference category: Other[1])	0.97	0.83*	1.03
	(0.87–1.07)	(0.71–0.97)	(0.91–1.18)
Education (ISCED-97):			
- Basic education (Reference category: Upper secondary)	1.06	1.59	0.72+
	(0.71–1.58)	(0.78–3.25)	(0.50–1.02)
- Lower secondary	0.89	0.92	0.88
	(0.76–1.03)	(0.70–1.19)	(0.73–1.06)
- Post secondary	1.06	1.11	0.98
	(0.88–1.26)	(0.85–1.45)	(0.78–1.25)
- Higher tertiary	1.23***	1.13	1.29***
	(1.09–1.38)	(0.97–1.31)	(1.11–1.51)
- Upper tertiary	0.79+	0.89	0.55*
	(0.60–1.03)	(0.66–1.21)	(0.31–0.95)
Currently not smoking (Reference category: currently smoking)	1.17**	1.11	1.15+
	(1.04–1.31)	(0.94–1.30)	(0.98–1.34)
Weight category:			

- Underweight (Reference category: normal weight)	1.04	0.51*	1.07
	(0.69–1.56)	(0.30–0.86)	(0.69–1.64)
- Overweight	0.90*	0.93	0.90
	(0.81–0.99)	(0.81–1.08)	(0.78–1.05)
- Obesity	0.99	0.97	1.04
	(0.86–1.13)	(0.81–1.17)	(0.86–1.26)
Number of chronic diseases	1.20***	1.29***	1.13***
	(1.15–1.26)	(1.19–1.39)	(1.08–1.18)
Activities of daily living (climbing stairs):			
- Slightly affected (Reference category: Not at all affected)	1.34***	1.34**	1.30**
	(1.18–1.53)	(1.11–1.62)	(1.09–1.54)
- Greatly affected	1.73***	1.37*	1.96***
	(1.43–2.10)	(1.04–1.81)	(1.54–2.50)
Activities of daily living (coping with everyday tasks)	1.25***	1.21*	1.30**
- Slightly affected (Reference category: Not at all affected)	(1.10–1.42)	(1.01–1.46)	(1.10–1.53)
	1.55***	1.41**	1.75***
- Greatly affected	(1.30–1.84)	(1.11–1.79)	(1.38–2.21)
Constant	1.23	0.75	1.88**
	(0.93–1.63)	(0.52–1.07)	(1.24–2.84)
Observations	3338	1711	1627
Pseudo R^2	0.120	0.115	0.140

[1]: married and living apart; widowed; divorced; never married; civil partnership, living apart; registered partner deceased; civil partnership dissolved. Incidence rate ratios were reported; confidence intervals in parentheses; *** p<0.001, ** p<0.01, * p<0.05, + p<0.10

Table S4. Determinants of seeing the doctor (for several reasons). Results of logistic regressions (1 = yes, visiting the doctor for this reason; 0 = otherwise) (also displaying the coefficients for the covariates).

		Reason for Doctor Visit: Chronic Illness–Men	Reason for Doctor Visit: Felt Unwell–Men	Reason for Doctor Visit: Requesting Advice–Men	Reason for Doctor Visit: Visit to the Doctor's Office (Without Consulting the Doctor)–Men	Reason for Doctor Visit: Preventive Medical Check-up/Vaccination – Men	Reason for Doctor Visit: Acute Illness–Women	Reason for Doctor Visit: Chronic Illness – Women	Reason for Doctor Visit: Felt Unwell–Women	Reason for Doctor Visit: Requesting Advice–Women	Reason for Doctor Visit: Visit to the Doctor's Office (Without Consulting the Doctor)–Women	Reason for Doctor Visit: Preventive Medical Check-up/Vaccination–Women
Materialistic mixed type (Reference category: Materialist)	1.47	1.32	0.86	0.60	0.73	0.95	1.14	0.60*	0.82	1.17	1.23	1.79*
	(0.83–2.59)	(0.75–2.31)	(0.35–2.12)	(0.32–1.14)	(0.42–1.29)	(0.56–1.61)	(0.73–1.76)	(0.37–0.95)	(0.45–1.49)	(0.66–2.09)	(0.76–1.99)	(1.12–2.84)
Post-materialistic mixed type	1.30	1.22	0.79	0.70	0.63	0.80	1.03	0.44***	0.76	0.93	1.35	2.25***
	(0.74–2.28)	(0.69–2.14)	(0.32–1.96)	(0.37–1.30)	(0.36–1.11)	(0.47–1.35)	(0.67–1.59)	(0.28–0.71)	(0.42–1.39)	(0.52–1.68)	(0.84–2.18)	(1.43–3.56)
Post-materialist	1.13	1.24	1.12	0.82	0.76	0.93	1.23	0.48**	0.60	1.08	1.19	1.63*
	(0.63–2.02)	(0.69–2.23)	(0.45–2.80)	(0.43–1.56)	(0.42–1.38)	(0.54–1.60)	(0.79–1.91)	(0.29–0.78)	(0.31–1.15)	(0.59–1.97)	(0.72–1.97)	(1.02–2.62)
Covariates:												
Age	0.96***	1.03***	0.99	1.01+	1.01*	1.01*	0.97***	1.02***	0.99+	1.00	1.00	1.01
	(0.95–0.97)	(1.02–1.04)	(0.97–1.00)	(1.00–1.02)	(1.00–1.03)	(1.00–1.02)	(0.96–0.98)	(1.01–1.04)	(0.97–1.00)	(0.99–1.01)	(0.99–1.01)	(1.00–1.01)
Marital status:												
Married, living together with spouse/partner (Reference category: Other[1])	1.19	0.79	1.10	1.06	1.12	1.21	0.82	0.94	0.82	0.99	1.00	0.98
	(0.87–1.63)	(0.57–1.09)	(0.63–1.92)	(0.71–1.58)	(0.78–1.62)	(0.89–1.64)	(0.63–1.06)	(0.69–1.27)	(0.55–1.23)	(0.69–1.42)	(0.74–1.34)	(0.76–1.27)
Education (ISCED-97):												
- Basic education (Reference category: Upper secondary)	1.10	1.89	3.34		1.14	2.31	1.00	2.53	1.06	1.68	0.22	0.79
	(0.29–4.15)	(0.48–7.35)	(0.63–17.73)		(0.22–5.84)	(0.62–8.54)	(0.33–3.07)	(0.75–8.47)	(0.22–4.99)	(0.45–6.27)	(0.03–1.74)	(0.22–2.89)
- Lower secondary	1.05	0.78	1.37	1.64	1.11	0.53	1.07	1.15	1.08	1.08	0.79	0.79
	(0.58–1.90)	(0.39–1.59)	(0.53–3.52)	(0.80–3.35)	(0.55–2.26)	(0.24–1.16)	(0.69–1.66)	(0.71–1.86)	(0.58–2.02)	(0.61–1.90)	(0.49–1.28)	(0.50–1.24)
- Post secondary	1.31	0.93	1.28	0.44	0.44	2.01*	1.25	1.16	1.30	1.00	0.97	1.21
	(0.72–2.38)	(0.44–1.96)	(0.43–3.84)	(0.13–1.48)	(0.15–1.28)	(1.11–3.65)	(0.78–2.02)	(0.61–2.22)	(0.62–2.74)	(0.50–2.00)	(0.55–1.72)	(0.75–1.94)
- Higher tertiary	1.15	0.85	1.13	1.35	0.81	1.56**	1.09	1.16	1.14	0.85	0.76	1.33*

	(0.85–1.57)	(0.62–1.18)	(0.65–1.95)	(0.91–2.01)	(0.57–1.16)	(1.16–2.09)	(0.81–1.48)	(0.82–1.65)	(0.72–1.83)	(0.55–1.29)	(0.54–1.06)	(1.00–1.77)
- Upper tertiary	1.50	0.77	2.02	1.12	0.91	1.13	0.69	0.41	1.40	0.82	0.45	1.93
	(0.59–3.78)	(0.30–1.96)	(0.55–7.42)	(0.39–3.19)	(0.34–2.45)	(0.48–2.68)	(0.14–3.37)	(0.04–3.68)	(0.17–11.54)	(0.10–6.63)	(0.06–3.66)	(0.54–6.92)
Currently not smoking (Reference category: currently smoking)	0.90	0.99	1.37	0.93	1.12	0.99	0.87	0.93	0.90	1.34	1.35	1.23
	(0.67–1.22)	(0.70–1.40)	(0.77–2.42)	(0.62–1.41)	(0.76–1.65)	(0.73–1.35)	(0.65–1.18)	(0.64–1.36)	(0.56–1.43)	(0.84–2.15)	(0.92–1.97)	(0.90–1.69)
Weight category:												
- Underweight (Reference category: normal weight)	0.68	0.76	1.17	2.85			2.12*	1.29	0.76	0.21	0.92	0.79
	(0.11–4.40)	(0.09–6.18)	(0.11–12.60)	(0.47–17.33)			(1.03–4.36)	(0.46–3.65)	(0.22–2.64)	(0.03–1.54)	(0.36–2.36)	(0.37–1.73)
- Overweight	1.23	0.97	0.88	0.56**	1.10	1.09	1.11	1.21	0.88	1.03	1.15	0.92
	(0.90–1.68)	(0.69–1.37)	(0.51–1.54)	(0.37–0.85)	(0.75–1.62)	(0.80–1.47)	(0.82–1.50)	(0.85–1.71)	(0.54–1.41)	(0.68–1.55)	(0.82–1.62)	(0.68–1.23)
- Obesity	0.89	1.48+	0.87	0.81	1.23	0.77	0.82	1.30	1.08	0.85	1.21	0.92
	(0.60–1.32)	(0.99–2.21)	(0.44–1.70)	(0.50–1.31)	(0.79–1.93)	(0.52–1.14)	(0.56–1.20)	(0.87–1.95)	(0.63–1.84)	(0.51–1.43)	(0.81–1.81)	(0.63–1.33)
Number of chronic diseases	0.94	1.61***	1.14	0.94	1.37***	0.95	1.09	1.77***	1.12	1.00	1.28***	0.89*
	(0.83–1.07)	(1.41–1.83)	(0.94–1.39)	(0.80–1.10)	(1.20–1.56)	(0.84–1.07)	(0.98–1.20)	(1.57–1.99)	(0.97–1.30)	(0.87–1.15)	(1.15–1.43)	(0.80–0.99)
Activities of daily living (climbing stairs):												
- Slightly affected (Reference category: Not at all affected)	0.93	1.42+	1.09	0.96	1.38	1.05	1.08	1.54*	0.88	0.90	1.22	0.85
	(0.63–1.38)	(0.96–2.09)	(0.57–2.09)	(0.58–1.58)	(0.89–2.13)	(0.72–1.53)	(0.76–1.53)	(1.04–2.27)	(0.52–1.51)	(0.54–1.49)	(0.83–1.82)	(0.60–1.19)
- Greatly affected	0.99	2.02*	0.82	2.18+	1.28	1.04	0.90	1.50	0.76	1.69	1.27	0.97
	(0.52–1.89)	(1.13–3.61)	(0.31–2.16)	(1.00–4.78)	(0.67–2.45)	(0.56–1.93)	(0.52–1.57)	(0.85–2.65)	(0.35–1.66)	(0.81–3.51)	(0.71–2.27)	(0.55–1.69)
Activities of daily living (coping with everyday tasks)												
- Slightly affected (Reference category: Not at all affected)	1.05	1.10	2.11*	1.22	1.16	0.91	1.11	1.13	1.63+	1.10	1.00	0.75+
	(0.74–1.49)	(0.75–1.61)	(1.11–3.99)	(0.77–1.92)	(0.76–1.78)	(0.64–1.29)	(0.79–1.56)	(0.76–1.68)	(0.96–2.75)	(0.68–1.78)	(0.68–1.48)	(0.54–1.04)
- Greatly affected	0.81	1.15	3.30**	0.50+	0.99	0.66	1.44	1.88*	2.15*	0.89	0.75	0.61+
	(0.46–1.43)	(0.67–1.96)	(1.41–7.71)	(0.23–1.09)	(0.54–1.80)	(0.38–1.16)	(0.86–2.41)	(1.09–3.24)	(1.03–4.49)	(0.43–1.84)	(0.42–1.31)	(0.36–1.02)
Constant	3.60*	0.02***	0.03***	0.09***	0.07***	0.16**	4.49**	0.04***	0.25+	0.08***	0.05***	0.21**
	(1.18–10.96)	(0.01–0.07)	(0.00–0.20)	(0.02–0.35)	(0.02–0.26)	(0.05–0.47)	(1.68–12.03)	(0.01–0.12)	(0.06–1.08)	(0.02–0.31)	(0.02–0.15)	(0.08–0.57)
Observations	1170	1170	1170	1159	1163	1163	1213	1213	1213	1213	1213	1213
Pseudo R^2	0.095	0.179	0.040	0.034	0.086	0.033	0.061	0.268	0.025	0.015	0.043	0.041

[1]: married and living apart; widowed; divorced; never married; civil partnership, living apart; registered partner deceased; civil partnership dissolved. Odds ratios were reported; confidence intervals in parentheses; *** p<0.001, ** p<0.01, * p<0.05, + p<0.10.

Table S5. Determinants of frequency of physician visits (total sample and stratified by sex). Results of negative binomial regressions (with 'Post-materialist' as reference category).

	(1)	(2)	(3)
	Frequency of Physician Visits–Total Sample	Frequency of Physician Visits–Men	Frequency of Physician Visits–Women
Materialist (Reference category: Post-materialist)	1.20*	0.91	1.40**
	(1.01–1.43)	(0.72–1.15)	(1.12–1.75)
Materialistic mixed type	1.15*	1.08	1.22*
	(1.02–1.30)	(0.92–1.26)	(1.03–1.45)
Post-materialistic mixed type	1.07	1.04	1.12
	(0.95–1.20)	(0.88–1.22)	(0.95–1.31)
Covariates	✓	✓	✓
Constant	0.92	0.72*	1.22
	(0.74–1.13)	(0.54–0.95)	(0.90–1.65)
Observations	3338	1711	1627
Pseudo R^2	0.053	0.058	0.056

Incidence rate ratios were reported; confidence intervals in parentheses; *** p<0.001, ** p<0.01, * p<0.05, + p<0.10; Covariates include age, marital status, education, chronic conditions, number of impairments in activities of daily living, smoking behavior, and weight category.

Table S6. Determinants of seeing the doctor (for several reasons). Results of logistic regressions (1 = yes, visiting the doctor for this reason; 0 = otherwise) (with 'Post-materialist' as reference category).

	**Reason for Doctor Visit: Acute Illness–Men**	**Reason for Doctor Visit: Chronic Illness–Men**	**Reason for Doctor Visit: Felt Unwell–Men**	**Reason for Doctor Visit: Requesting Advice–Men**	**Reason for Doctor Visit: Visit to the Doctor's Office (Without Consulting the Doctor)–Men**	**Reason for Doctor Visit: Preventive Medical Check-up/Vaccination–Men**	**Reason for Doctor Visit: Acute Illness–Women**	**Reason for Doctor Visit: Chronic Illness–Women**	**Reason for Doctor Visit: Felt Unwell–Women**	**Reason for Doctor Visit: Requesting Advice–Women**	**Reason for Doctor Visit: Visit to the Doctor's Office (Without Consulting the Doctor)–Women**	**Reason for Doctor Visit: Preventive Medical Check-up/Vaccination–Women**
Materialist (Reference category: Post-materialist)	0.87	0.82	0.91	1.25	1.30	1.09	0.81	2.08**	1.68	0.93	0.85	0.61*
	(0.49–1.55)	(0.46–1.47)	(0.36–2.25)	(0.66–2.37)	(0.72–2.34)	(0.63–1.86)	(0.52–1.26)	(1.27–3.41)	(0.87–3.23)	(0.51–1.71)	(0.51–1.41)	(0.38–0.98)
Materialistic mixed type	1.30	1.07	0.77	0.74	0.96	1.03	0.92	1.25	1.37	1.09	1.04	1.09
	(0.91–1.85)	(0.72–1.57)	(0.42–1.43)	(0.46–1.18)	(0.63–1.47)	(0.72–1.45)	(0.65–1.29)	(0.83–1.87)	(0.78–2.39)	(0.68–1.75)	(0.70–1.55)	(0.78–1.52)
Post-materialistic mixed type	1.14	0.99	0.71	0.86	0.82	0.86	0.84	0.92	1.28	0.87	1.14	1.37+
	(0.81–1.60)	(0.67–1.44)	(0.39–1.31)	(0.55–1.34)	(0.54–1.26)	(0.61–1.21)	(0.61–1.17)	(0.61–1.38)	(0.74–2.20)	(0.54–1.39)	(0.78–1.67)	(1.00–1.88)
Covariates	✓	✓	✓	✓	✓	✓	✓	✓	✓	✓	✓	✓
Constant	3.16***	0.04***	0.05***	0.12***	0.04***	0.18***	2.43**	0.03***	0.13***	0.08***	0.09***	0.35**
	(1.69–5.91)	(0.02–0.08)	(0.01–0.15)	(0.05–0.28)	(0.02–0.09)	(0.10–0.36)	(1.28–4.59)	(0.01–0.06)	(0.05–0.35)	(0.03–0.22)	(0.04–0.19)	(0.18–0.67)
Observations	1170	1170	1170	1159	1163	1163	1214	1214	1214	1214	1214	1214
Pseudo R^2	0.094	0.179	0.040	0.033	0.086	0.033	0.057	0.267	0.0250	0.015	0.042	0.041

Odds ratios were reported; confidence intervals in parentheses; *** p<0.001, ** p<0.01, * p<0.05, + p<0.10; Covariates include age, marital status, education, chronic conditions, number of impairments in activities of daily living, smoking behavior, and weight category.

Table S7. Determinants of frequency of physician visits (total sample and stratified by sex). Results of negative binomial regressions (main model extended by (log) household net equivalent income).

	(1)	(2)	(3)
	Frequency of Physician Visits–Total Sample	Frequency of Physician Visits–Men	Frequency of Physician Visits–Women
Materialistic mixed type (Reference category: Materialist)	0.96	1.18	0.88
	(0.81–1.13)	(0.93–1.50)	(0.70–1.09)
Post-materialistic mixed type	0.89	1.14	0.80*
	(0.75–1.05)	(0.89–1.44)	(0.65–0.99)
Post-materialist	0.83*	1.10	0.72**
	(0.70–0.99)	(0.87–1.39)	(0.57–0.90)
Covariates	✓	✓	✓
Constant	1.23	0.74	1.80*
	(0.86–1.76)	(0.47–1.17)	(1.09–3.00)
Observations	3332	1708	1624
Pseudo R^2	0.053	0.058	0.055

Incidence rate ratios were reported; confidence intervals in parentheses; *** p<0.001, ** p<0.01, * p<0.05, + p<0.10; Covariates include age, marital status, education, income, chronic conditions, number of impairments in activities of daily living, smoking behavior, and weight category.

Table S8. Determinants of seeing the doctor (for several reasons). Results of logistic regressions (1 = yes, visiting the doctor for this reason; 0 = otherwise). (main model extended by (log) household net equivalent income).

	Reason for Doctor Visit: Acute Illness– Men	Reason for doctor visit: Chronic illness – Men	Reason for doctor visit: Felt unwell– Men	Reason for doctor visit: Requesting advice– Men	Reason for doctor visit: Visit to the doctor's office (without consulting the doctor)– Men	Reason for doctor visit: Preventive medical check-up/vaccination – Men	Reason for Doctor Visit: Acute Illness– Women	Reason for doctor visit: Chronic illness – Women	Reason for Doctor Visit: Felt Unwell– Women	Reason for Doctor Visit: Requesting Advice– Women	Reason for Doctor Visit: Visit to the Doctor's Office (Without Consulting the Doctor)– Women	Reason for Doctor Visit: Preventive Medical Check-up/Vaccination – Women
Materialistic mixed type (Reference category: Materialist)	1.47	1.32	0.86	0.60	0.73	0.95	1.14	0.60*	0.82	1.17	1.23	1.79*
	(0.83–2.59)	(0.75–2.31)	(0.35–2.12)	(0.32–1.14)	(0.42–1.29)	(0.56–1.61)	(0.73–1.76)	(0.37–0.95)	(0.45–1.49)	(0.66–2.09)	(0.76–1.99)	(1.12–2.84)
Post-materialistic mixed type	1.30	1.22	0.79	0.70	0.63	0.80	1.03	0.44***	0.76	0.93	1.35	2.25***
	(0.74–2.28)	(0.69–2.14)	(0.32–1.96)	(0.37–1.30)	(0.36–1.11)	(0.47–1.35)	(0.67–1.59)	(0.28–0.71)	(0.42–1.39)	(0.52–1.68)	(0.84–2.18)	(1.43–3.56)
Post-materialist	1.13	1.24	1.12	0.82	0.76	0.93	1.23	0.48**	0.60	1.08	1.19	1.63*
	(0.63–2.02)	(0.69–2.23)	(0.45–2.80)	(0.43–1.56)	(0.42–1.38)	(0.54–1.60)	(0.79–1.91)	(0.29–0.78)	(0.31–1.15)	(0.59–1.97)	(0.72–1.97)	(1.02–2.62)
Covariates	✓	✓	✓	✓	✓	✓	✓	✓	✓	✓	✓	✓
Constant	3.60*	0.02***	0.03***	0.09***	0.07***	0.16**	4.49**	0.04***	0.25+	0.08***	0.05***	0.21**
	(1.18–10.96)	(0.01–0.07)	(0.00–0.20)	(0.02–0.35)	(0.02–0.26)	(0.05–0.47)	(1.68–12.03)	(0.01–0.12)	(0.06–1.08)	(0.02–0.31)	(0.02–0.15)	(0.08–0.57)
Observations	1170	1170	1170	1159	1163	1163	1213	1213	1213	1213	1213	1213
Pseudo R^2	0.095	0.179	0.040	0.034	0.086	0.033	0.061	0.268	0.025	0.015	0.043	0.041

Odds ratios were reported; confidence intervals in parentheses; *** p<0.001, ** p<0.01, * p<0.05, + p<0.10; Covariates include age, marital status, education, income, chronic conditions, number of impairments in activities of daily living, smoking behavior, and weight category.

Table S9. Determinants of frequency of physician visits (total sample and stratified by sex). Results of negative binomial regressions (main model extended by employment status).

	**(1)**	**(2)**	**(3)**
	Frequency of Physician Visits–Total Sample	Frequency of Physician Visits–Men	Frequency of Physician Visits–Women
Materialistic mixed type (Reference category: Materialist)	0.95	1.16	0.88
	(0.80–1.13)	(0.92–1.47)	(0.71–1.09)
Post-materialistic mixed type	0.89	1.14	0.80*
	(0.75–1.06)	(0.90–1.45)	(0.65–0.99)
Post-materialist	0.84*	1.11	0.72**
	(0.71–1.00)	(0.87–1.41)	(0.58–0.90)
Covariates	✓	✓	✓
Constant	1.11	0.67*	1.70**
	(0.86–1.44)	(0.48–0.93)	(1.18–2.45)
Observations	3337	1710	1627
Pseudo R^2	0.054	0.060	0.056

Incidence rate ratios were reported; confidence intervals in parentheses; *** p<0.001, ** p<0.01, * p<0.05, + p<0.10; Covariates include age, marital status, education, employment status, chronic conditions, number of impairments in activities of daily living, smoking behavior, and weight category.

Table S10. Determinants of seeing the doctor (for several reasons). Results of logistic regressions (1 = yes, visiting the doctor for this reason; 0 = otherwise). (main model extended by employment status).

	**Reason for Doctor Visit: Acute Illness–Men**	**Reason for Doctor Visit: Chronic Illness – Men**	**Reason for doctor visit: Felt unwell–Men**	**Reason for Doctor Visit: Requesting Advice–Men**	**Reason for doctor visit: Visit to the doctor's office (without consulting the doctor)–Men**	**Reason for doctor visit: Preventive medical check-up/vaccination – Men**	**Reason for Doctor Visit: Acute Illness–Women**	**Reason for Doctor Visit: Chronic Illness – Women**	**Reason for Doctor visit: Felt Unwell–Women**	**Reason for Doctor Visit: Requesting Advice–Women**	**Reason for Doctor Visit: Visit to the Doctor's Office (Without Consulting the Doctor)–Women**	**Reason for Doctor Visit: Preventive Medical Check-up/Vaccination – Women**
Materialistic mixed type (Reference category: Materialist)	1.48	1.30	0.89	0.57+	0.74	0.95	1.13	0.60*	0.79	1.22	1.28	1.75*
	(0.84–2.62)	(0.74–2.28)	(0.36–2.20)	(0.30–1.08)	(0.42–1.30)	(0.56–1.60)	(0.73–1.75)	(0.37–0.95)	(0.43–1.45)	(0.68–2.18)	(0.79–2.08)	(1.10–2.80)
Post-materialistic mixed type	1.30	1.22	0.78	0.69	0.64	0.80	1.04	0.44***	0.74	0.97	1.40	2.21***
	(0.74–2.29)	(0.69–2.14)	(0.31–1.92)	(0.37–1.29)	(0.36–1.12)	(0.47–1.35)	(0.67–1.60)	(0.27–0.71)	(0.41–1.36)	(0.54–1.76)	(0.86–2.26)	(1.40–3.49)
Post-materialist	1.14	1.24	1.09	0.82	0.77	0.92	1.23	0.48**	0.58	1.12	1.23	1.61*
	(0.64–2.03)	(0.69–2.23)	(0.44–2.72)	(0.43–1.55)	(0.43–1.40)	(0.54–1.58)	(0.79–1.92)	(0.29–0.78)	(0.30–1.11)	(0.61–2.07)	(0.74–2.05)	(1.00–2.59)
Covariates	✓	✓	✓	✓	✓	✓	✓	✓	✓	✓	✓	✓
Constant	2.59*	0.03***	0.03***	0.17***	0.05***	0.21***	1.95+	0.05***	0.21**	0.08***	0.08***	0.21***
	(1.18–5.67)	(0.01–0.08)	(0.01–0.14)	(0.06–0.46)	(0.02–0.14)	(0.09–0.46)	(0.96–3.96)	(0.02–0.12)	(0.07–0.61)	(0.03–0.23)	(0.03–0.18)	(0.10–0.44)
Observations	1169	1169	1169	1158	1162	1162	1214	1214	1214	1214	1214	1214
Pseudo R^2	0.095	0.181	0.048	0.035	0.086	0.033	0.058	0.267	0.028	0.023	0.047	0.043

Odds ratios were reported; confidence intervals in parentheses; *** p<0.001, ** p<0.01, * p<0.05, + p<0.10; Covariates include age, marital status, education, employment status, chronic conditions, number of impairments in activities of daily living, smoking behavior, and weight category.

Table S11. Determinants of frequency of physician visits (total sample and stratified by sex). Results of negative binomial regressions (main model extended by monthly dummies).

	(1)	(2)	(3)
	Frequency of physician visits – Total sample	Frequency of physician visits – Men	Frequency of physician visits – Women
Materialistic mixed type (Reference category: Materialist)	0.96	1.20	0.88
	(0.81–1.14)	(0.95–1.52)	(0.71–1.09)
Post-materialistic mixed type	0.89	1.15	0.81*
	(0.75–1.05)	(0.91–1.45)	(0.66–1.00)
Post-materialist	0.84*	1.11	0.73**
	(0.70–0.99)	(0.88–1.40)	(0.58–0.90)
Covariates	✓	✓	✓
Constant	1.03	0.67+	1.42+
	(0.76–1.40)	(0.43–1.05)	(0.95–2.14)
Observations	3338	1711	1627
Pseudo R^2	0.054	0.060	0.056

Incidence rate ratios were reported; confidence intervals in parentheses; *** p<0.001, ** p<0.01, * p<0.05, + p<0.10; Covariates include age, monthly dummies, marital status, education, chronic conditions, number of impairments in activities of daily living, smoking behavior, and weight category.

Table S12. Determinants of seeing the doctor (for several reasons). Results of logistic regressions (1 = yes, visiting the doctor for this reason; 0 = otherwise). (main model extended by monthly dummies).

	**Reason for Doctor Visit: Acute Illness– Men**	**Reason for Doctor Visit: Chronic Illness – Men**	**Reason for Doctor Visit: Felt Unwell– Men**	**Reason for Doctor Visit: Requestin g Advice– Men**	**Reason for Doctor Visit: Visit to the Doctor's Office (Without Consulting the Doctor)– Men**	**Reason for Doctor Visit: Preventive Medical Check-up/Vaccina tion – Men**	**Reason for Doctor Visit: Acute Illness– Women**	**Reason for Doctor Visit: Chronic Illness – Women**	**Reason for Doctor Visit: Felt Unwell– Women**	**Reason for Doctor Visit: Requestin g Advice– Women**	**Reason for Doctor Visit: Visit to the Doctor's Office (Without Consulting the Doctor)– Women**	**Reason for Doctor Visit: Preventive Medical Check-up/Vaccina tion – Women**
Materialistic mixed type (Reference category: Materialist)	1.48	1.24	0.82	0.57+	0.76	0.98	1.15	0.61*	0.80	1.16	1.21	1.80*
	(0.84–2.61)	(0.71–2.18)	(0.33–2.04)	(0.30–1.09)	(0.43–1.34)	(0.58–1.67)	(0.74–1.78)	(0.38–0.97)	(0.44–1.47)	(0.65–2.07)	(0.74–1.97)	(1.13–2.87)
Post-materialistic mixed type	1.27	1.19	0.78	0.69	0.66	0.81	1.04	0.45***	0.75	0.91	1.36	2.31***
	(0.72–2.24)	(0.68–2.09)	(0.31–1.92)	(0.37–1.29)	(0.37–1.17)	(0.48–1.37)	(0.67–1.60)	(0.28–0.72)	(0.41–1.37)	(0.50–1.64)	(0.84–2.19)	(1.46–3.65)
Post-materialist	1.13	1.18	1.09	0.81	0.78	0.93	1.25	0.49**	0.57+	1.08	1.12	1.65*
	(0.63–2.02)	(0.65–2.12)	(0.43–2.71)	(0.42–1.54)	(0.43–1.42)	(0.54–1.60)	(0.80–1.95)	(0.30–0.81)	(0.30–1.11)	(0.58–1.98)	(0.67–1.86)	(1.02–2.65)
Covariates	✓	✓	✓	✓	✓	✓	✓	✓	✓	✓	✓	✓
Constant	3.71*	0.02***	0.07**	0.16**	0.02***	0.10***	2.18+	0.03***	0.32+	0.10***	0.10***	0.09***
	(1.34–10.27)	(0.01–0.08)	(0.01–0.38)	(0.04–0.59)	(0.00–0.07)	(0.03–0.29)	(0.90–5.27)	(0.01–0.08)	(0.09–1.11)	(0.03–0.36)	(0.03–0.27)	(0.03–0.25)
Observations	1170	1165	1170	1154	1163	1163	1206	1214	1214	1214	1214	1214
Pseudo R^2	0.099	0.187	0.050	0.040	0.100	0.037	0.059	0.273	0.031	0.022	0.050	0.050

Odds ratios were reported; confidence intervals in parentheses; *** p<0.001, ** p<0.01, * p<0.05, + p<0.10; Covariates include age, monthly dummies, marital status, education, chronic conditions, number of impairments in activities of daily living, smoking behavior, and weight category.

Table S13. Determinants of frequency of physician visits (total sample and stratified by sex). Results of negative binomial regressions (main model extended by town size).

	(1)	(2)	(3)
	Frequency of Physician Visits – Total Sample	Frequency of Physician Visits – Men	Frequency of Physician Visits – Women
Materialistic mixed type (Reference category: Materialist)	0.96	1.20	0.86
	(0.81–1.13)	(0.95–1.50)	(0.70–1.07)
Post-materialistic mixed type	0.90	1.16	0.81*
	(0.77–1.07)	(0.92–1.46)	(0.65–0.99)
Post-materialist	0.83*	1.09	0.72**
	(0.70–0.98)	(0.87–1.37)	(0.58–0.90)
Covariates	✓	✓	✓
Constant	1.06	0.63*	1.63*
	(0.76–1.49)	(0.40–0.97)	(1.10–2.44)
Observations	3338	1711	1627
Pseudo R^2	0.056	0.060	0.061

Incidence rate ratios were reported; confidence intervals in parentheses; *** p<0.001, ** p<0.01, * p<0.05, + p<0.10; Covariates include age, town size, marital status, education, chronic conditions, number of impairments in activities of daily living, smoking behavior, and weight category.

Table S14. Determinants of seeing the doctor (for several reasons). Results of logistic regressions (1 = yes, visiting the doctor for this reason; 0 = otherwise) (main model extended by town size).

	**Reason for Doctor Visit: Acute Illness–Men**	**Reason for Doctor Visit: Chronic Illness – Men**	**Reason for Doctor Visit: Felt Unwell–Men**	**Reason for Doctor Visit: Requesting Advice–Men**	**Reason for Doctor Visit: Visit to the Doctor's Office (Without Consulting the Doctor)–Men**	**Reason for Doctor Visit: Preventive Medical Check-up/Vaccination – Men**	**Reason for Doctor Visit: Acute Illness–Women**	**Reason for Doctor Visit: Chronic Illness – Women**	**Reason for Doctor Visit: Felt Unwell–Women**	**Reason for Doctor Visit: Requesting Advice–Women**	**Reason for Doctor Visit: Visit to the Doctor's Office (Without Consulting the Doctor)–Women**	**Reason for Doctor Visit: Preventive Medical Check-up/Vaccination – Women**
Materialistic mixed type (Reference category: Materialist)	1.43	1.30	0.86	0.59	0.73	0.95	1.13	0.60*	0.83	1.19	1.23	1.81*
	(0.81–2.53)	(0.74–2.29)	(0.35–2.12)	(0.31–1.12)	(0.41–1.28)	(0.56–1.61)	(0.73–1.76)	(0.37–0.96)	(0.45–1.51)	(0.66–2.12)	(0.76–2.01)	(1.13–2.88)
Post-materialistic mixed type	1.28	1.21	0.79	0.68	0.63	0.79	1.04	0.45***	0.76	0.93	1.33	2.29***
	(0.72–2.25)	(0.69–2.14)	(0.32–1.94)	(0.36–1.28)	(0.36–1.11)	(0.46–1.33)	(0.67–1.60)	(0.28–0.72)	(0.42–1.39)	(0.51–1.69)	(0.82–2.16)	(1.45–3.62)
Post-materialist	1.13	1.21	1.07	0.80	0.76	0.93	1.24	0.48**	0.61	1.08	1.19	1.62*
	(0.63–2.02)	(0.67–2.18)	(0.43–2.67)	(0.42–1.52)	(0.42–1.38)	(0.54–1.60)	(0.79–1.94)	(0.29–0.79)	(0.31–1.18)	(0.59–1.99)	(0.71–1.97)	(1.01–2.61)
Covariates	✓	✓	✓	✓	✓	✓	✓	✓	✓	✓	✓	✓
Constant	2.20+	0.03***	0.03***	0.16**	0.07***	0.22**	1.44	0.05***	0.13**	0.08***	0.09***	0.24**
	(0.88–5.51)	(0.01–0.08)	(0.01–0.16)	(0.05–0.51)	(0.02–0.20)	(0.09–0.54)	(0.62–3.33)	(0.02–0.13)	(0.03–0.48)	(0.03–0.28)	(0.04–0.25)	(0.10–0.56)
Observations	1170	1170	1170	1159	1163	1163	1214	1214	1214	1214	1214	1214
Pseudo R^2	0.0985	0.183	0.0494	0.0476	0.0959	0.0403	0.0612	0.276	0.0341	0.0235	0.0548	0.0475

Odds ratios were reported; confidence intervals in parentheses; *** p<0.001, ** p<0.01, * p<0.05, + p<0.10; Covariates include age, town size, marital status, education, chronic conditions, number of impairments in activities of daily living, smoking behavior, and weight category.

Table S15. Determinants of frequency of physician visits (total sample and stratified by sex). Results of negative binomial regressions (main model extended by town size).

	(1)	(2)	(3)	(4)	(5)	(6)
	Frequency of Physician Visits – Total Sample Aged 18 to 49 Years	Frequency of Physician Visits – Men Aged 18 to 49 Years	Frequency of Physician Visits – Women Aged 18 to 49 Years	Frequency of Physician Visits – Total Sample Aged 50+ Years	Frequency of Physician Visits – Men Aged 50+ Years	Frequency of Physician Visits – Women Aged 50+ Years
Materialistic mixed type (Reference category: Materialist)	0.90	1.59*	0.71*	0.98	0.99	1.00
	(0.69–1.17)	(1.11–2.27)	(0.51–0.98)	(0.79–1.21)	(0.73–1.34)	(0.75–1.32)
Post-materialistic mixed type	0.87	1.43*	0.73*	0.90	1.01	0.82
	(0.67–1.13)	(1.01–2.02)	(0.54–1.00)	(0.72–1.12)	(0.74–1.39)	(0.62–1.09)
Post-materialist	0.82	1.35+	0.64**	0.83	0.98	0.72*
	(0.63–1.06)	(0.95–1.92)	(0.47–0.87)	(0.67–1.04)	(0.72–1.34)	(0.54–0.97)
Covariates	✓	✓	✓	✓	✓	✓
Constant	1.08	0.50*	1.96**	1.17	0.72	2.02
	(0.74–1.59)	(0.28–0.89)	(1.21–3.19)	(0.66–2.09)	(0.36–1.42)	(0.86–4.77)
Observations	1640	827	813	1698	884	814
Pseudo R^2	0.054	0.043	0.072	0.050	0.058	0.051

Incidence rate ratios were reported; confidence intervals in parentheses; *** p<0.001, ** p<0.01, * p<0.05, + p<0.10; Covariates include age, marital status, education, chronic conditions, number of impairments in activities of daily living, smoking behavior, and weight category.
